# Jaw exercise therapy for the treatment of trismus in head and neck Cancer: a prospective three-year follow-up study

**DOI:** 10.1007/s00520-020-05517-7

**Published:** 2020-11-24

**Authors:** Ove Karlsson, Therese Karlsson, Nina Pauli, Paulin Andréll, Caterina Finizia

**Affiliations:** 1 Department of Anaesthesiology, Institute of Clinical Science, Sahlgrenska Academy at the University of Gothenburg, Sahlgrenska University Hospital, Gothenburg, Sweden; 2 Department of Otorhinolaryngology, Region Västra Götaland, Sahlgrenska University Hospital, Gothenburg, Sweden; 3 Department of Otorhinolaryngology, Head and Neck Surgery, Institute of Clinical Sciences, Sahlgrenska Academy at the University of Gothenburg, Sahlgrenska University Hospital, Gothenburg, Sweden; 4 Department of Molecular and Clinical Medicine/Pain Center, Institute of Medicine, Sahlgrenska Academy at the University of Gothenburg, Göteborg, Sweden

**Keywords:** Trismus, Radiotherapy, Health-related quality of life, Exercise therapy, Mouth opening ability

## Abstract

**Purpose:**

This study aims to examine effects of jaw exercise on trismus 3 years following completion of a post-radiotherapy jaw exercise intervention.

**Methods:**

Prospective study including 50 patients with head-and-neck cancer receiving radiotherapy and/or chemotherapy, plus a matched control group. The intervention group underwent 10 weeks of jaw exercise training. Patients were followed pre-and postintervention and 3 years postintervention completion. Outcome measures were maximal interincisal opening (MIO), trismus-related symptoms, and health-related quality-of-life as measured by Gothenburg Trismus Questionnaire, EORTC QLQ-C30, and EORTC QLQ-H&N35.

**Results:**

The intervention group had a statistically significantly higher mean MIO compared with the control group (40.1 mm and 33.9 mm, respectively, *p* < 0.001), reported less trismus-related problems and had an improved health-related quality-of-life when compared with the control group at the 3-year follow-up. These differences were all statistically significant.

**Conclusion:**

Jaw exercise therapy resulted in increased MIO, less trismus-related symptoms, and improved health-related quality-of-life. Jaw exercise therapy should be initiated early, in a structured manner and continued long-term.

## Introduction

Trismus is defined as reduced mouth opening, Maximum Interincisal Opening (MIO) ≤ 35 mm [1], and can be caused by benign disorders, infections in the oral cavity, trauma, and malignant disorders [2]. Common symptoms associated with trismus besides limited mouth opening are pain, difficulties with chewing and swallowing, and poor oral hygiene. Subsequently, trismus has a negative impact on both mental health and health-related quality of life (HRQL) [2].

In patients with head and neck cancer (HNC), trismus is a common symptom with a reported incidence after oncologic treatment of up to 40% [3]. The majority of the HNC patients are treated surgically or with radiotherapy and sometimes with additional chemotherapy for advanced stage tumors according to Swedish Cancer Guidelines [4].

Symptom trajectory reveals a typical worsening of the condition immediately after treatment, peaking 6–9 months posttreatment [5]. Spontaneous trismus recovery can be expected to a certain degree, but for the majority of afflicted patients, status remains unchanged due to the posttreatment development of tissue fibrosis in the masticatory muscles and temporomandibular joint. There is no standardized treatment for radiotherapy-induced trismus, and although several studies have assessed exercise intervention, these studies included small study populations [6–8]. A recently published study by this research group reported that structured exercise intervention with jaw mobilizing devices improved mouth opening, trismus-related symptoms, and HRQL at 2 years following completion of jaw exercise training [9].

However, data for longer term follow-up is lacking. Hence, this study aims to investigate the effects of mouth opening, trismus-related symptoms, and HRQL after structured mouth opening intervention in HNC patients with trismus at 3 years following jaw exercise therapy.

## Material and methods

### Participants

Patients with newly diagnosed HNC tumors and location expected to develop trismus were included in the study between 2007 and 2012 from five medical centers in the Västra Götaland region, Sweden (Table 1). Inclusion criteria were radiation therapy with or without chemotherapy and development of trismus after oncologic treatment.

**Table 1:** Patient characteristics in the intervention group and the control group at baseline

	Intervention group *n = 50	Control group *n = 50
	Mean (range)	Mean (range)
Age mean (range)	57.9 (30–75)	58.0 (29–80)
	*n* (%)	*n* (%)
Gender		
Male	31 (62)	31 (62)
Female	19 (38)	19 (38)
Treatment regimen		
Radiotherapy only	7 (14)	8 (16)
Radiochemotherapy	39 (80)	38 (76)
Radiotherapy + surgery	4 (8)	4 (8)
Tumor location		
Oropharynx	38 (76)	38 (76)
Tumor colli	6 (12)	6 (12)
Oral cavity	1 (2)	1 (2)
Nasopharynx	5 (10)	5 (10)
Staging		
1	1 (2)	0 (0)
2	8 (18)	4 (9)
3	8 (18)	12 (27)
4	27 (61)	28 (64)
ACE-27		
No comorbidity	29 (58)	20 (40)
Mild comorbidity	13 (26)	18 (36)
Moderate comorbidity	7 (14)	10 (20)
Severe comorbidity	1 (2)	2 (4)

*ACE-27*, Adult Comorbidity Evaluation

*There was no statistically significant difference (on any variable) between the intervention group and the control group

Patients with difficulties filling out questionnaires, edentulous patients, and patients with poor general health (including but not limited to for instance dementia or substance abuse) were excluded as well as patient undergoing surgical treatment and who had trismus prior to starting treatment. Patients residing in Gothenburg underwent regular clinical evaluation by one single oral surgeon at the Department of Oral and Maxillofacial Surgery, Institute of Odontology and Public Dental Service, Gothenburg, Sweden and those who developed trismus were invited to participate in a 10-week intervention program performed by the same oral surgeon [7].

The control group consists of patients living outside the Gothenburg catchment area and was matched according to tumor location, tumor stage, gender, age (within a 5-year interval), comorbidity, and radiation dose. The control group was followed up with appointments according to local guidelines and MIO was measured by the local hospital dentist. However, no structured intervention program for trismus existed during the study period. Any attempt of improving mouth opening, structured or otherwise, was registered by the study coordinator [7].

### Outcome measures, assessment, and questionnaires

The primary endpoint in this study was the MIO measured in millimeters, which was measured using a ruler with the patient sitting in an upright position. MIO was measured as the maximal distance between the edges of the incisors of the mandible and the maxilla, expressed in millimeters. Patients were assessed before and after exercise intervention and after 2 and 3 years, respectively. Secondary endpoints were HRQL and trismus-related symptoms assessed by Patient-Reported Outcome Measures (PROM) instruments, including The European Organization for Research and Treatment of Cancer Core Questionnaire (EORTC QLQ-C30) and the related module for Head and Neck Questionnaire in EORTC (EORTC QLQ-HN35) and Gothenburg Trismus Questionnaire (GTQ) [10–12].

The patients’ comorbidity was assessed according to the *Adult Comorbidity Evaluation 27* (ACE-27) [13–15]. Tumors were classified and staged according to the TNM system of classification of malignant tumors determined by the Union for International Cancer Control [16].

### Trismus intervention

**Fig. 1:**
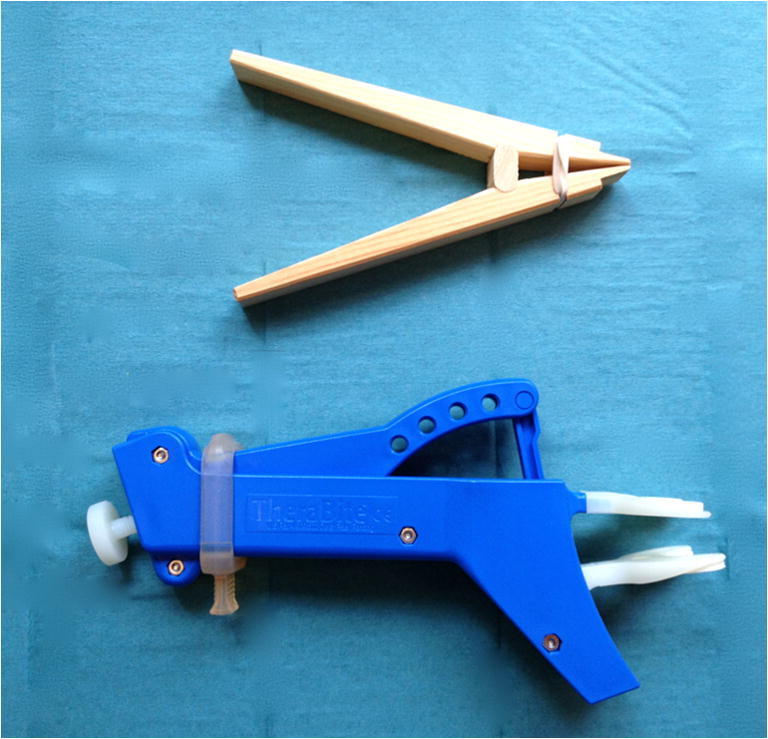
Top, Engström jaw device. Bottom, Therabite® jaw device

Patients in the intervention group and the 50 matched controls were enrolled 3–6 months after receiving radiotherapy. The structured trismus exercise was performed with a jaw mobilizing device during 10 weeks. The devices used in this study were the TheraBite® jaw device or the Engström jaw device, Fig. 1 [7]. A study by Pauli et al. showed no significant difference between the Therabite® and Engström jaw mobilizers at 3 months postintervention and is considered to be equivalent devices [17]. All patients in the intervention group received written and oral instructions as well as a demonstration of the device by the same oral surgeon. The patients started the exercise with warm-up movements and stretching for 30 s, if possible, using a jaw mobilizing device five times daily. The exercise consisted of active movements, biting against resistance, and passive, stretch movements of the jaw. MIO was evaluated by the same oral surgeon before and after intervention and thereafter at 1, 2, and 3 years postintervention. After the 10-week of exercise intervention, the patients were instructed to continue the exercise at least three times weekly or more often if needed. A more precise definition of the exercise program is outlined in Pauli et al., 2014 [7].

### Patient-reported outcome

#### EORTC QLQ-C30 and EORTC QLQ-H&N35

The EORTC QLQ-C30 is a cancer-specific questionnaire that assesses HRQL in patients with cancer [18]. For functional domains and the global quality of life domain, scores range from 0 to 100, where a high score is equal to a high level of functioning or a high level of global quality of life. For single items, the scores also range from 0 to 100, but a higher score is indicative of a higher symptom burden. To address additional symptoms associated specifically with HNC, a complementary 35-item module was used, the EORTC QLQ-H&N35. As described before in symptom scales and single items, a score of 100 indicates the worst possible symptoms and 0 indicates no symptoms [10, 11] [19].

#### Gothenburg trismus questionnaire (GTQ)

The GTQ is a trismus-specific self-administered questionnaire, well accepted by patients and has shown good validity and reliability [12]. It is composed of three domains containing the following 13 items: jaw-related problems (six items), eating limitations (four items), and muscular tension (three items). The remaining eight items are retained as single items addressing facial pain and pain associated with trismus and if trismus is affecting work, leisure, or social life. The domains and single items range from 0 to 100, where 100 equates to maximum symptomatology and 0 represents no symptoms.

## Statistical methods

Descriptive statistics were calculated according to standard procedures. For comparison between groups, Fisher’s exact test was used for dichotomous variables and the Mantel-Haenszel X^2^ exact test was used for ordered categorical variables and X^2^ exact test was used for non-ordered categorical variables. For comparison between groups, the Mann–Whitney U test was used for continuous variables. Continuous variables are reported using mean and confidence intervals. All tests are two-tailed and conducted at a 5% significance level.

## Results

### Patient characteristics

Patient characteristics are presented in Table 1. Both the intervention group and the control group developed trismus within 9 months from radiotherapy termination of which the majority presented with trismus within 3–6 months. In the intervention group (*n* = 50), three patients were lost at 3-year follow-up (deceased *n* = 2, unspecified *n* = 1). In the control group (*n* = 50), seven patients were lost at 3-year follow-up (deceased *n* = 6, unspecified *n* = 1).

### Jaw exercise therapy

The intervention group and the control group trained to varying degrees during the follow-up period. At 3-year follow-up, 41 of the 47 (87%) intervention patients no longer had trismus (i.e., > 35 mm) and 32 of 47 (68%) had continued performing the exercises. Of the patients still exercising at 3-year follow-up, 26 of 32 (81%) no longer had trismus. The exercise frequency in the intervention group was as follows: one trained occasionally, 24 trained once a day, and seven trained two to four times a day. A total of 19 out of 32 (59%) estimated their training as very effective. In the control group, 23 of the 43 (53%) patients no longer had trismus at 3-year follow-up. Fourteen of 43 (33%) patients were exercising, and seven of 14 (50%) no longer had trismus. The exercise frequency in the control group was as follows: three trained occasionally, seven trained once a day, and four trained two to four times a day, with four of 14 (29%) estimated their training as very effective.

### Maximum interincisal opening (MIO)

**Fig. 2:**
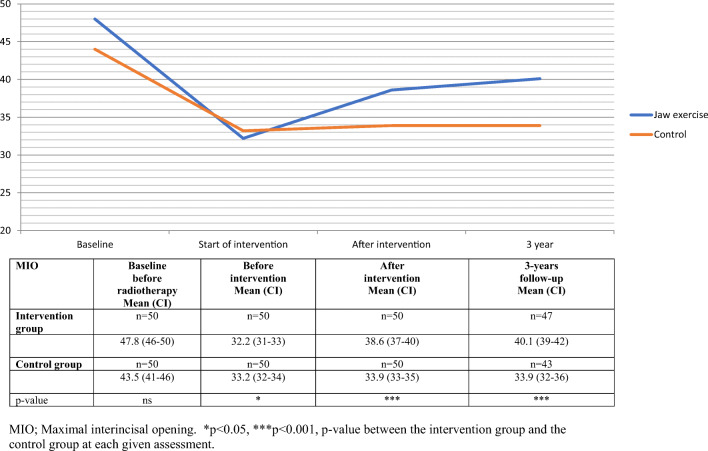
Maximal interincisal opening, mean value, and 95% confidence interval (CI) for head and neck cancer patients over time, from baseline to 3-year follow-up. MIO, maximal interincisal opening. *p < 0.05, ***p < 0.001, *p* value between the intervention group and the control group at each given assessment

At the 3-year follow-up, the intervention group had a statistically significant improved mean MIO (40.1 mm), compared with the control group (33.9 mm) *p* < 0.001, Fig. 2.

### Gothenburg trismus questionnaire (GTQ)

After exercise intervention, the intervention group reported a statistically significant improvement in three domains, *jaw-related problems*, *eating limitations*, *and muscular tension* as well as in the questions about facial pain right now, compared with the control group (Table 2). At the 3-year follow-up, the intervention group reported statistically different improvements in all domains and items of the GTQ questionnaire.

**Table 2:** GTQ-score for head and neck cancer patients before and after intervention and 3-year follow-up

	Before intervention			After intervention			36-months follow-up		
GTQ	I, *n* = 50 mean (CI)	C, *n* = 50 mean (CI)	p	I, *n* = 50 mean (CI)	C, *n* = 50 mean (CI)	p	I, *n* = 47 mean (CI)	C, *n* = 43 mean (CI)	*p*
Jaw-related problems	41.4 (36–47)	41.5 (35–48)	ns	22.9 (17–29)	43.1 (37–49)	***	9.0 (6–12)	46.0 (39–53)	***
Eating limitations	46.5 (37–56)	40.0 (33–47)	ns	28.1 (21–35)	39.5 (33–46)	*	4.9 (3–7)	46.4 (38–55)	***
Muscular tension	26.3 (22–31)	23.8 (18–29)	ns	13.2 (10–17)	27.5 (22–33)	***	7.8 (5–11)	40.3 (33–47)	***
Facial pain right now	24.3 (18–31)	20.7 (14–27)	ns	9.0 (4–14)	20.7 (15–26)	***	2.5 (0–4)	20.2 (15–26)	***
Facial pain worst last month	43.0 (36–51)	40.3 (33–48)	ns	22.7 (16–29)	30.7 (24–38)	ns	5.0 (3–7)	27.5 (21–34)	***
Facial pain average	38.3 (32–45)	35.3 (28–43)	ns	21.0 (15–27)	30.0 (23–37)	ns	5.0 (2–7)	25.6 (19–32)	***
Facial pain social, leisure, and family activities	24.0 (16–32)	23.5 (16–31)	ns	15.0 (7–23)	20.0 (13–27)	ns	0.5 (0–2)	20.9 (14–28)	***
Facial pain affecting ability to work	25.0 (17–33)	23.5 (15–32)	ns	13.5 (6–21)	21.0 (14–28)	*	1.1 (0–3)	18.0 (12–25)	***
Limitation in opening mouth	49.0 (43–55)	45.0 (36–54)	ns	33.0 (26–40)	40.0 (33–47)	ns	15.4 (11–20)	43.0 (34–52)	***
LOM social, leisure and family activities	24.0 (18–30)	24.5 (17–32)	ns	16.5 (8–25)	26.5 (20–33)	**	2.7 (0–5)	22.1 (15–29)	***
LOM affecting ability to work	24.5 (16–33)	25.0 (17–33)	ns	14.0 (6–22)	22.0 (15–30)	*	2.1 (0–5)	22.1 (15–29)	***

*ns*, not statistically significant; *I*, intervention group; *C*, control group; *GTQ*, Gothenburg Trismus Questionnaire; *CI*, 95% confidence interval; *HNC*, head and neck cancer; Domains and single items range 0–100, where 100 indicate maximal amount of symptoms and 0 is equal to no symptoms. *P* values for analysis of statistical significant difference in mean scores between the intervention group and the control group, before and after intervention and at 3-year follow-up

******p < 0.05, *p* < 0.01, *p* < 0.001

### EORTC QLQ-C30

Immediately after exercise intervention, the control group and intervention group reported similar scores, with the only statistically significant difference noted in *Global quality of life*. However, at the 3-year follow-up, the intervention group had improved in all functional domains and most single items compared with the control group, resulting in statistical differences between the groups in 14 out of 15 items (Table 3). The greatest improvement was found in the domain *Global quality of life* (∆ 28).

**Table 3:** EORTC QLQ-C30-score for trismus head and neck cancer patients before and after intervention and at 3-year follow-up, and reference data from 3 years head and neck cancer survivors and a normative age-and sex-matched sample

	Before intervention			After intervention			36-months follow-up			HNC3 #	Norm #
Functional scales	I, *n* = 50 Mean (CI)	C, *n* = 50 Mean (CI)	p	I, *n* = 50 Mean (CI)	C, *n* = 50 Mean (CI)	p	I, *n* = 47 Mean (CI)	C, *n* = 43 Mean (CI)	p	*n* = 133 Mean (SD)	*n* = 562 Mean (SD)
Physical function	73.9 (69–79)	72.7 (66–79)	ns	83.7 (80–88)	79.6 (74–85)	ns	90.1 (86–94)	76.4 (70–83)	**	85.6 (21.2)	88.9 (18.1)
Role function	46.0 (36–56)	52.3 (43–61)	ns	61.7 (53–71)	63.7 (55–72)	ns	92.9 (89–97)	61.6 (51–72)	***	83.3 (30.1)	86.5 (24.8)
Emotional function	70.3 (64–77)	72.0 (65–79)	ns	82.9 (77–88)	75.5 (68–83)	ns	85.5 (81–90)	71.9 (64–80)	**	85.0 (18.7)	84.6 (20.1)
Cognitive function	76.0 (71–82)	76.0 (68–84)	ns	82.7 (76–89)	75.3 (68–83)	ns	86.9 (82–92)	75.2 (67–83)	*	87.0 (16.5)	86.9 (18.1)
Social function	56.7 (48–65)	61.0 (52–70)	ns	70.7 (63–79)	67.3 (59–76)	ns	91.8 (88–96)	66.7 (56–77)	***	86.0 (22.2)	89.5 (20,6)
Global QL	54.2 (49–60)	58.0 (53–63)	ns	66.3 (61–72)	57.2 (51–63)	*	82.6 (79–86)	49.8 (42–57)	***	73.2 (21.3)	76.5 (22.0)
Fatigue	49.6 (43–57)	45.0 (37–53)	ns	34.0 (26–42)	36.3 (29–44)	ns	18.7 (13–24)	38.5 (29–48)	***	22.8 (22.9)	19.7 (21.4)
Nausea/vom	15.7 (10–21)	13.0 (7–19)	ns	9.3 (3–16)	8.33 (3–13)	ns	2.8 (1–5)	13.6 (6–21)	**	3.1 (9.0)	3.3 (11.0)
Pain − 25.5	36.3 (28–45)	36.0 (29–44)	ns	21.7 (15–29)	27.0 (20–34)	ns	11.0 (7–15)	28.3(21–36)	***	14.9 (20.7)	17.7 (24.6)
Dyspnea	36.7 (29–45)	37.3 (29–46)	ns	24.0 (18–30)	25.3 (18–33)	ns	15.6 (10–21)	31.0 (21–41)	*	17.8 (23.9)	16.5 (24.1)
Insomnia	24.0 (17–31)	32.7 (24–42)	ns	23.3 (17–30)	21.1 (14–29)	ns	17.7 (10–25)	35.7 (25–46)	**	18.8 (26.4)	16.5 (24.9)
Appetite loss	49.3 (40–59)	46.3 (37–56)	ns	36.1 (26–46)	35.3 (26–45)	ns	12.1 (5–19)	29.3 (19–40)	**	11.7 (25.1)	3.4 (12.7)
Constipation	26.0 (17–35)	24.0 (16–32)	ns	14.0 (6–22)	16.7 (9–25)	ns	11.3 (4–19)	14.0 (7–21)	ns	10.8 (21.9)	5.0 (16.0)
Diarrhea	10.7 (5–16)	12.0 (6–18)	ns	17.0 (9–25)	10.0 (5–16)	ns	7.1 (2–12)	16.3 (9–24)	*	3.8 (11.4)	5.1 (15.9)
Financial Problems	19.3 (12–26)	30.0 (20–40)	ns	13.3 (6–20)	29.3 (19–40)	*	9.2 (2–16)	31.8 (20–44)	**	11.6 (25.2)	6.6 (19.7)

*ns*, not statistically significant; *I*, intervention group; *C*, control group; *CI*, 95% confidence interval; *EORTC QLQ C30*, European Organization for Research and Treatment of Cancer Quality of Life Questionnaire-Core 30; Score range 0–100 points. High scores for a functional scale and the global quality of life scale represent high level of functioning or high level of global quality of life. High scores for a single item represent high level of symptoms. *P* values for analysis of statistical significant difference in mean scores between the intervention group and the control group, before and after intervention and at 3-year follow-up. ^#^HNC3 = 3 years HNC patients survivors and norm = a normative age- and sex-matched sample [19].

******p < 0.05, *p* < 0.01,*p* < 0.001

### EORTC QLQ-H&N35

Only *mouth opening* differed significantly between the groups postintervention. However, at the 3-year follow-up, the intervention group reported higher HRQL in 13 out of 14 items compared with the control group (Table 4).

**Table 4:** EORTC QLQ-H&N35-score for head and neck cancer patients before and after intervention and 3-year follow-up, and reference data from 3 years head and neck cancer survivors and a normative age-and sex-matched sample

EORTC QLQ-H&N35	Before intervention			After intervention			36-months follow-up			HNC3 #	Norm #
Symptom scales	I, *n* = 50 Mean (CI)	C, *n* = 50 Mean (CI)	p	I, *n* = 50 Mean (CI)	C, *n* = 50 Mean (CI)	p	I, *n* = 47 Mean (CI)	C, *n* = 43 Mean (CI)	p	*n* = 133 Mean (SD)	*n* = 562 Mean (SD)
Local pain	5	39.4 (32–47)	ns	30.0 (23–37)	33.8 (27–41)	ns	15.4 (11–20)	28.9 (22–36)	**	14.5 (19.8)	3.0 (9.4)
Swallowing	43.6 (36–51)	38.8 (31–47)	ns	32.3 (25–39)	35.4 (27–44)	ns	18.4 (12–24)	38.5 (30–47)	***	10.9 (19.6)	2.0 (7.2)
Senses	37.0 (30–44)	39.7 (32–48)	ns	26.7 (19–34)	32.0 (25–39)	ns	17.0 (11–23)	32.2 (24–40)	**	20.5 (27.4)	5.8 (16.1)
Speech	30.9 (24–38)	28.0 (21–35)	ns	19.1 (13–25)	25.4 (19–32)	ns	9.2 (5–14)	28.9 (21–37)	***	12.1 (19.1)	6.1 (13.2)
Social eating	45.4 (37–54)	42.3 (35–50)	ns	31.8 (25–38)	42.3 (34–50)	ns	14.9 (10–20)	39.9 (31–49)	***	11.7 (22.6)	2.7 (10.0)
Social contact	19.6 (12–27)	16.4 (10–22)	ns	10.0 (5–15)	16.0 (10–23)	ns	2.7 (1–4)	24.5 (17–32)	***	7.6 (14.7)	3.8 (10.6)
Sexuality	56.6 (47–66)	59.7 (49–71)	ns	45.1 (34–56)	47.3 (37–58)	ns	25.0 (17–33)	44.6 (34–55)	*	28.8 (36.3)	26.1 (33.6)
Teeth problems	24.0 (16–32)	24.7 (16–33)	ns	20.7 (13–29)	29.9 (21–39)	ns	12.1 (6–18)	35.7 (25–47)	***	21.4 (32.3)	10.1 (21.4)
Opening mouth	44.0 (37–51)	45.3 (37–54)	ns	30.7 (21–40)	45.3 (37–54)	**	15.6 (10–21)	46.5 (36–57)	***	17.6 (29.3)	1.8 (10.8)
Dry mouth	79.3 (72–87)	82.7 (75–91)	ns	71.3 (63–80)	78.2 (70–87)	ns	52.2 (42–62)	75.2 (67–84)	***	47.3 (36.3)	12.3 (22.3)
Sticky saliva	66.7 (56–77)	69.4 (60–79)	ns	54.7 (45–65)	62.6 (53–73)	ns	32.6 (25–41)	55.8 (46–65)	***	18.6 (28.4)	6.9 (17.5)
Coughing	32.0 (22–42)	30.0 (21–39)	ns	28.0 (19–37)	32.0 (22–42)	ns	17.7 (12–24)	31.8 (22–42)	*	17.3 (24.6)	16.8 (24.4)
Feeling ill	31.3 (22–41)	29.3 (21–37)	ns	20.7 (12–29)	22.9 (14–31)	ns	11.3 (6–17)	30.2 (21–40)	**	10.8 (20.0)	11.3 (21.5)
Pain killers	67.3 (54–81)	62.0 (48–76)	ns	42.0 (28–56)	40.0 (26–54)	ns	27.7 (14–41)	44.2 (26–62)	ns	No data	No data

*ns*, not statistically significant; *I*, intervention group; *C*, control group; *CI*, 95% confidence interval; *EORTC QLQ-H&N35*, European Organization for Research and Treatment of Cancer Quality of Life Questionnaire Head and Neck 35; *HNC*, head and neck cancer. ^#^ HNC3 = HNC patients survivors at 3 years, Norm = a normative age- and sex-matched sample [19]. Score range 0–100 points. High scores for a single item represent high level of symptoms. *P* values for analysis of statistical significant difference in mean scores between the intervention group and the control group, before and after intervention and at 3-year follow-up

*p < 0.05, **p < 0.01, ***p < 0.001

## Discussion

This, to date, longest prospective study investigating the effects of jaw exercise in irradiated HNC patients with trismus found that patients receiving early and structured jaw exercise therapy had a significantly higher MIO, reported significantly less trismus-related symptom (as measured by GTQ), and had a higher HRQL compared with the control group at 3 years follow-up.

A recent review by Kamstra et al. found 211 articles originating from 20 studies of trismus and the effects of jaw exercise therapy [20]. The review found that 12 studies appeared to have some effect, but due to various limitations, the authors concluded that no clinical guidelines could be given following the review. A recurring issue is that patients are not evaluated long-term making conclusions of efficacy difficult to draw. In this study, however, with the longest follow-up to date, 87% of patients receiving structured jaw exercise therapy no longer had trismus at 3 years (i.e., MIO ≤ 35 mm). Additionally, apart from reporting statistically significant better HRQL on all items in EORTC QLQ-H&N35 compared with the control group at the 3-year follow-up, the intervention group reported data comparable with healthy individuals (Table 3) regarding *social contact*, *sexuality*, and *teeth problems* [21]. The control group on the other hand, in fact reported worse HRQL in all questionnaire items when compared with the pooled HRQL-data of all HNC survivors at three years [21]. Thus, the negative impact of untreated trismus on HRQL is highlighted and this study finds that jaw exercise therapy appears effective long term for treating symptoms and improving HRQL.

A previous study by Pauli et al. reported the MIO, GTQ, and HRQL data for this cohort at 2 years posttraining intervention [9]. When comparing the 3-year data with the two-year follow-up by Pauli et al., some points are worth highlighting. Firstly, mouth opening ability as measured by MIO appears unchanged for both the intervention and control group between the two time-points. Additionally, the symptom burden recorded by GTQ also remains stable. However, in contrast, HRQL according to EORTC QLQ-C30 continues to improve in the intervention group, with significant differences in an additional six domains/items (*physical function*, *emotional function*, *cognitive function*, *fatigue*, *nausea and vomiting*, *pain)* at 3 years compared with the control group, as opposed to significant difference in only three items (*role function*, *social function*, and *global quality of life*) at the two-year follow-up. A similar pattern was seen in EORTC QLQ-H&N35 where only four items (*speech*, *social contact*, *teeth problems*, and *mouth opening*) were significantly better in the intervention group at 2 years compared with the control group, whereas at 3 years statistically significant differences were found between the groups in all items. The static values of MIO long term may be explained by remaining fibrosis, whereas the improvement in HRQL despite unchanged MIO might reflect the long-term effects of reduced trismus on other aspects of life, which cannot be as instantly measured as MIO.

Effective jaw exercise training involves mainly three aspects highlighted in this study. Firstly, exercising early and in a structured manner when trismus first develops appears pivotal. This was concluded as despite 33% of the control group also ended up performing jaw exercise training due to problematic trismus (albeit unstructured), the mean MIO did not improve to the same extent as the intervention group. Moreover, only 53% of control group patients were free of trismus at 3 years as opposed to 87% in the intervention group. Lastly, many patients continued to exercise following the completion of the initial 10 weeks of intervention (68%), in contrast to other studies which have previously shown that adherence to independent and unsupervised training may be poor [22]. The high exercise compliance rate in this study may be explained by the immediate positive effect exercise has on mouth opening ability and more importantly, the regression of MIO if training is absent.

The strengths of this study lie in its prospective study design as it is the only long-term follow-up study post jaw exercise training for trismus patients including both objective and subjective measures. It also has a large cohort and a small drop-out rate. It is of course limited by not being randomized allowing for selection bias, albeit this is minimized by the inclusion of a carefully matched control group.

### Clinical implications

As trismus has a negative impact on function and HRQL, it is an important issue for clinicians to be aware of. This study has shown that jaw exercise therapy started early, in a structured manner and with continued training, appears to improve both MIO and HRQL—effects which appear stable up to 3 years. Therefore, early identification of these patients with enrollment in structured exercise programs is advocated in an attempt to counteract post-radiotherapy trismus.

## Conclusions

Jaw exercise therapy for irradiated HNC patients who develop trismus is effective up to 3 years after completion of the training and results in increased MIO, less trismus-related symptoms as well as improved HRQL. In order to maximize the results, jaw exercise therapy should be initiated early, in a structured manner and continued long term.
